# Supplementary material for: Single‐Cell Metabolic Imaging Reveals Glycogen‐Driven Adaptations in Endothelial Cells
Source: Adv Sci (Weinh). 2025 Nov 26;13(7):e08517. doi: 10.1002/advs.202508517 (PMC12866687; doi:10.1002/advs.202508517)
Supplement: Supplementary file 1 — Supporting Information [file ADVS-13-e08517-s001.docx]

**Supplementary Information**

**Single-Cell Metabolic Imaging Reveals Glycogen-Driven Adaptations in Endothelial Cells**

Rahuljeet S. Chadha^1^, Benjamin Yang^1^, Dongqiang Yuan^2^, Edmund D. Kapelczak^3^, Philip A. Kocheril^1^, Alonso Tapia^2^, Shivansh Mahajan^1^, Adrian Colazo^1^, Naseeb K. Malhi^2^, Joseph A. Ambarian^1^, Xuejing Liu^2^, Tara A. TeSlaa^3^, Zhen B. Chen^2^*, and Lu Wei^1^*

^1^Division of Chemistry and Chemical Engineering, California Institute of Technology, Pasadena, CA 91125, USA

^2^Department of Diabetes Complications and Metabolism, Beckman Research Institute, City of Hope Medical Center, Duarte, CA 91010, USA

^3^Department of Molecular and Medical Pharmacology, David Geffen School of Medicine at UCLA, Los Angeles, CA 90095, USA

*Email: [lwei@caltech.edu](mailto:lwei@caltech.edu) (L.W.), [zhenchen@coh.org](mailto:zhenchen@coh.org) (Z.B.C.)

**Figure S1. Chemometric approaches for glycogen phenotyping in ECs. (a)** SRS image targeting the –CH_3_ channel (protein) in ECs under hyperglycemic conditions; **(b)** Maximum intensity projection of a hSRS stack comprising 25 images acquired across 2067-2235 cm^-1^ with a step size of 7 cm^-1^ used for chemometric analysis; **(c)** Mapped glycogen image generated using LASSO (CD_Glycogen_); **(d)** Overlay of CH_3_, and CD_Glycogen_ image derived from LASSO analysis; **(e)** Mapped glycogen image generated using spectral phasor analysis (CD_Glycogen_). The inset shows the phasor plot where the boxed area (yellow) represents pixels attributed to glycogen; **(f)** Overlay of CH_3_, and CD_Glycogen_ image generated using spectral phasor analysis. Scale bar: 20 µm.

**Figure S2. Correlative PAS staining of glycogen pools in ECs. (a, b)** Representative SRS images targeted at the –CH_3_ (protein) and C–D (on–off) channels, respectively, for HT-treated HUVECs; **(c)** Bright field image of the same set of cells after PAS staining. White arrows represent glycogen pools. Scale bar: 20 µm.

**Figure S3. Glycogen pools in ECs are not an artifact of glucose isotopologues.
(a)** Representative SRS image targeted at the –CH_3_ (protein) for HT-treated HUVECs containing unlabeled glucose (h_7_-glucose); **(b)** Bright field image of the same set of cells after PAS staining; **(c)** C–D images (on–off) of the same corresponding field of views (FOVs). White arrows represent glycogen pools; fi-EV = fixation-induced extracellular vesicle. Scale bar: 20 µm.

**Figure S4. PFA fixation induces glycogen-enriched extracellular vesicles. (a)** Representative overlay image (C–D/CH_2_ channels) of fixed HUVECs treated with HT using d_7_-glucose for three days. Yellow arrow represents intracellular glycogen reserves, while the white arrow indicates the extracellular glycogen in fixation-induced EVs (fi-EVs) after PFA fixation; **(b)** Zoomed-in image of the boxed region in (a), targeted at the C–D channel (on–off) indicating glycogen-enriched fi-EV (white arrow); **(c)** Corresponding zoomed-in CH_2_ (lipid) image of the boxed region in (a); **(d)** Normalized SRS spectrum of intracellular and extracellular glycogen regions from (a). Scale bar: 20 µm.

**Figure S5. Raman spectra of glucose isotopologue, 6,6-d_2_-glucose.** Normalized spontaneous Raman spectrum of 100 mM solution (soln) of 6,6-d_2_-glucose in water (red circles) with corresponding fitted data (maroon solid line). Normalized SRS spectrum of subcellular glycogen from HT-treated ECs incubated with d_2_-glucose (red squares with solid line). Data are presented as mean ± SEM.

**Figure S6. GSK3 inhibition using an alternative small molecule inhibitor, SB-216763.** Representative SRS images targeting the –CH_3_ (protein) and C–D (on-off) channel for live HUVECs treated with HT+SB incubated with either d_7_-glucose, or d_2_-glucose. White arrowheads represent subcellular glycogen pools. Scale bar: 20 µm.


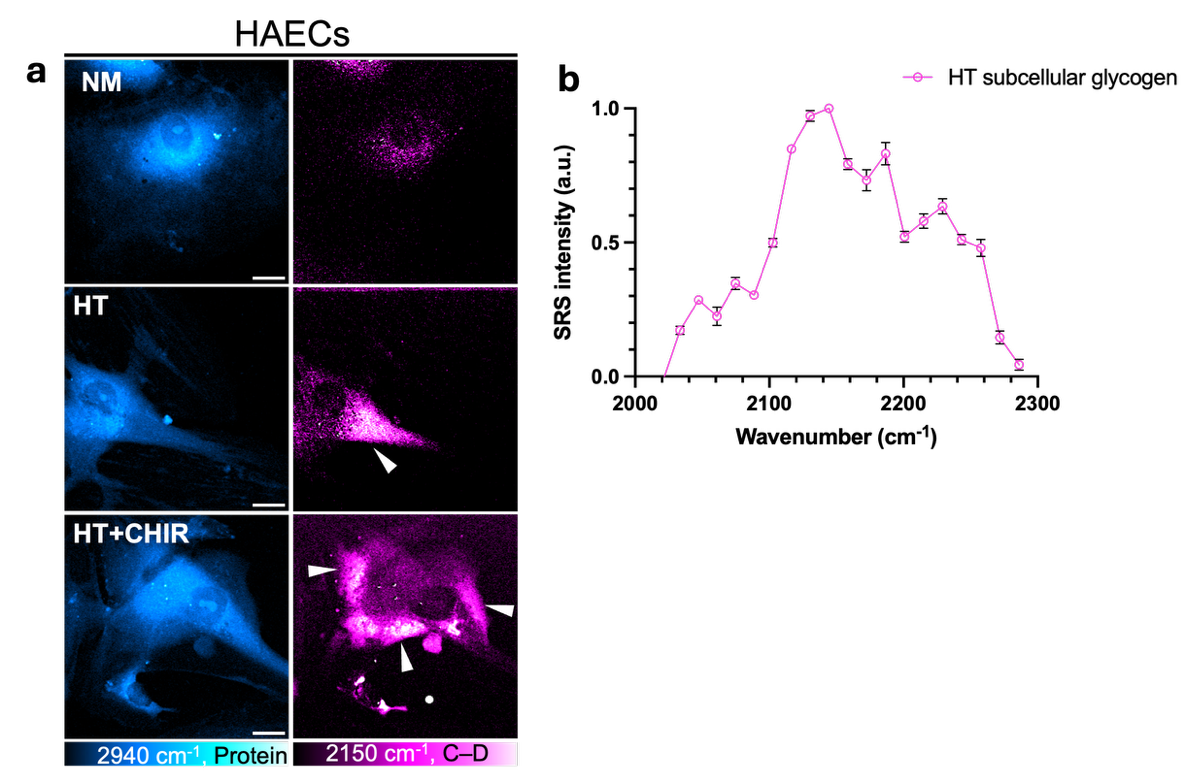
**Figure S7. Glycogen phenotyping in live human aortic endothelial cells (HAECs). (a)** SRS images targeted at the protein channel (–CH_3_, left) and C–D (on–off, right) channels for HAECs exposed to NM, HT, or HT+CHIR for 72 hr. White arrowheads indicate subcellular glycogen pools. Scale bar: 20 µm; **(b)** Hyperspectral SRS of the subcellular pools in HT-treated cells (panel a) confirms the chemical signature of glycogen. Data represented as mean ± SEM.

**Figure S8. Glycogen detection in bulk ECs using luminescence. (a)** Calibration curve for glycogen with different concentrations (µg/mL) measured by luminescence (rlu); **(b)** Glycogen concentration (µg/mL) in ECs treated with HT, or HT+CHIR for three days, quantified using the calibration curve in (a) (n=9 technical replicates from three independent experiments). Statistical significance was analyzed using two-tailed unpaired Student’s t-test, ****p<0.0001. Data are presented as mean ± SEM.

**Table S1. Glycogen concentration detected in bulk HUVECs using luminescence.** Mean concentration of glycogen (µg/mL) for the corresponding treatments detected using the luminescence assay (in Figure S7) and their standard error of mean (SEM). The analytical sensitivity (γ) of the assay is calculated to be 18,394 rlu/(µg/mL).


**a)**

**b)**

**Figure S9. Bioenergetics profiling of HUVECs**. **(a)** Extracellular acidification rate (ECAR) profile of ECs under NM, HT, or HT+CHIR conditions measured with the Seahorse assay; **(b)** Oxygen consumption rate (OCR) profile of endothelial cells (ECs) under the same conditions. Mitochondrial respiration modulators were sequentially injected (oligomycin, FCCP, rotenone, and antimycin A). Data represented as mean ± SEM.

**Figure S10**. **mRNA levels of eNOS, VE-cadherin and ICAM1 in NM-, HT-, or HT+CHIR-treated HUVECs for 3 days.** Data represented as mean ± SD. Statistical significance was analyzed using two-tailed unpaired Student’s t-tests. ***p<0.001, **p<0.01, *p<0.05.

**Figure S11**. **Correlative Mitochondrial staining (Fluorescence) and d_7_-glucose imaging (SRS) of NM-, HT-, and HT+CHIR-exposed live ECs**. Representative overlay images of fluorescence (MitoDeepRed) and SRS (C–D) of live HUVECs are shown. The bottom panel represents selected zoomed-in regions from the top panel (yellow box). White arrowheads indicate subcellular glycogen pools. Scale bar: 20 µm.

**Figure S12. Longitudinal imaging of glycogen metabolism in HUVECs.** SRS intensities of segmented glycogen C–D (on-off) per cell pulsed with HT, or HT+CHIR for 72 hr, and chased in glucose-free media for 48 hr. Data are presented as mean ± SEM from at least three independent experiments.

**Figure S13. Viability assay for HeLa and HUVEC under glucose starvation.** Relative viability of HeLa and HUVECs cultured in glucose-deficient media for 72 hours. Data are presented as mean ± SEM from three biological replicates.

**
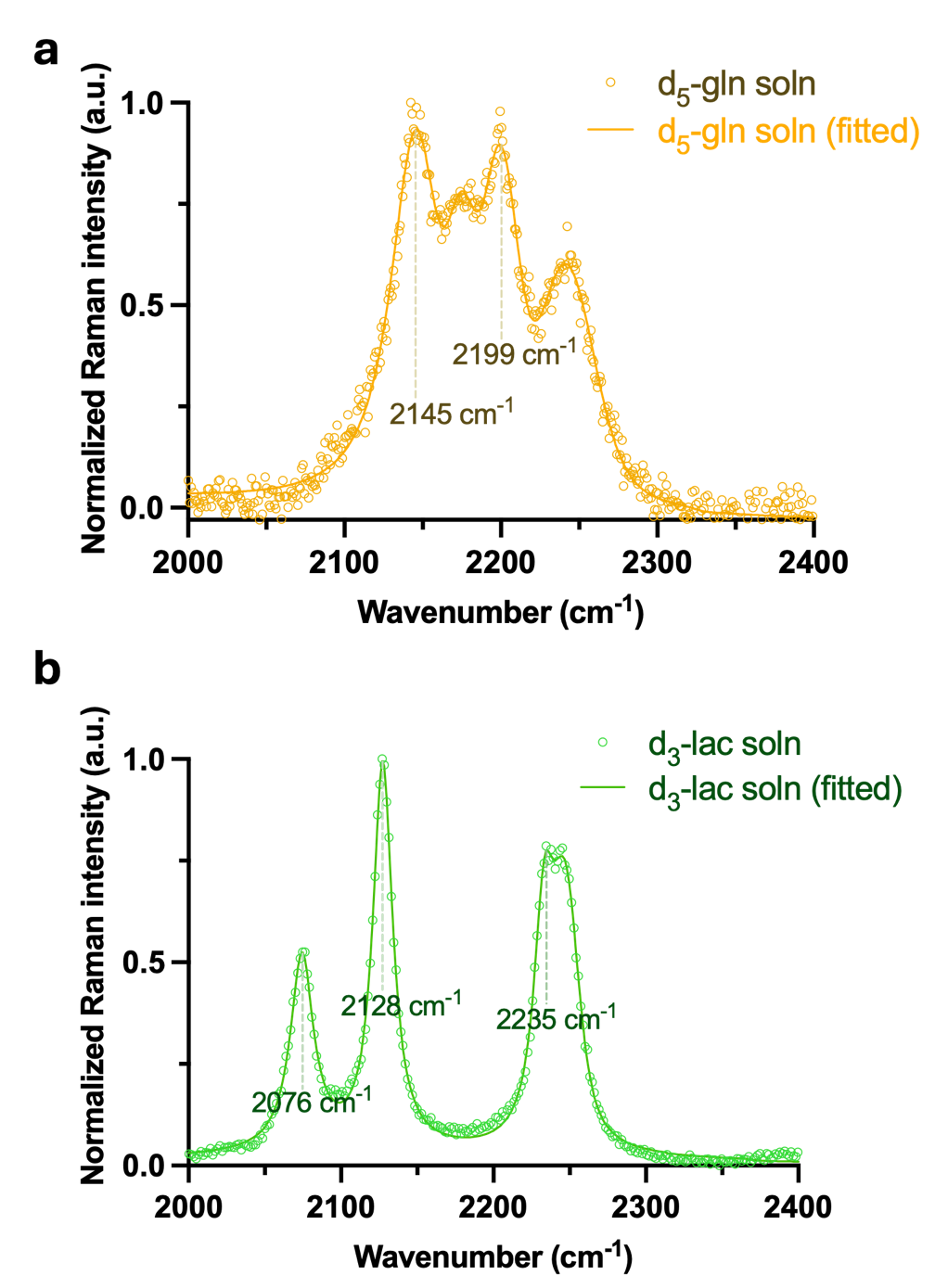
**

**Figure S14**. Spontaneous Raman spectra of **(a)** 200 mM of d_5_-glutamine solution (soln) in water (experimental, yellow circles; fitted, yellow solid line) and **(b)** 25 mM d_3_-lactate solution (soln) in water (experimental, green circles; fitted, green solid line).

**Figure S15.** **No differences in glutamine reliance upon glycogen depletion in ECs. (a)** Illustration of pulse-chase experiment. After 72 hr of corresponding treatments, HUVECs were starved for 24 hr in glucose-free media followed by a chase with 5 mM d_5_-gln in glucose-free media for 24 hr; **(b)** Representative SRS images at the Protein (–CH_3_) and C–D (on-off) channels. Scale bar: 20 µm; **(c)** Quantitative analysis of SRS signal of C–D bonds per cell (n=38, 42, 39 cells for NM, HT, and HT+CHIR, respectively). Data are presented as mean ± SEM. Statistical significance was analyzed using two-tailed unpaired Student’s t-tests, ns: no statistical difference.

**a**

**Figure S16.** **Presence of glycogen influences specific metabolic demands in HUVECs. (a)** Normalized spontaneous Raman spectrum of 50 mM d_5_-phenylalanine solution (soln) in water (experimental, blue circles; fitted, blue solid line); **(b)** Schematic of pulse-chase experiment with 5 mM d_5_-phenylalanine in glucose-free ECGM. **(c)** Representative SRS images at the Protein (–CH_3_) and C–D (on-off) channels. Scale bar: 20 µm; **(d)** Quantitative analysis of SRS signal of C–D bonds per cell (n=33, 25, 28 cells for NM, HT, and HT+CHIR, respectively). Data are presented as mean ± SEM. Statistical significance was analyzed using two-tailed unpaired Student’s t-tests, ns: no statistical difference.

**Figure S17.** **No differences in lactate reliance upon glycogen depletion. (a)** Schematic of pulse-chase experiment. After 72 hr of corresponding treatments, HUVECs were starved for 24 hr in glucose-free media followed by a chase with 30 mM d_3_-lac in glucose-free media for 24 hr; **(b)** Representative SRS images at the Protein (–CH_3_) and C–D (on-off) channels. Scale bar: 20 µm; **(c)** Quantitative analysis of SRS signal of C–D bonds per cell (n=16, 14, 11 cells for NM, HT, and HT+CHIR, respectively). Data are presented as mean ± SEM. Statistical significance was analyzed using two-tailed unpaired Student’s t-test, ns: no statistical difference.

**Figure S18. D_3_-lactate tracing in downstream metabolites of HUVECs**. MS-based quantification of C–D incorporation in **(a)** acetyl carnitine; **(b)** palmitoleic acid (C16:1); and **(c)** oleic acid (C18:1)**.** Data are presented as mean (weighted isotopologues) ± SEM from at least three independent experiments. Statistical significance was analyzed using one-way ANOVA tests. **p<0.01,*p<0.05. n = 3 biological replicates.

**a**

**b**

**c**

**Figure S19.** **Cellular viability of HUVECs treated with Raman probes.** Relative viability (%) of HUVECs incubated with deuterium-labeled Raman probes compared to cells with no probes: glucose (d_7_- vs h_7_-) for 72 hr, glutamine (d_5_- vs h_5_-) for 24 hr, or lactate (d_3_- vs. h_3_-) for 24 hr, normalized to the baseline regular glucose incubation for 24 hr (control). Data are presented as mean ± SEM*.* n = 3 biological replicates.

**Table S2**. **List of primer sequences used for qPCR.**

| **qPCR primers** | **Forward** | **Reverse** |
| --- | --- | --- |
| ICAM1 | GTGTCCTGTATGGCCCCCGACT | ACCTTGCGGGTGACCTCCCC |
| VCAM1 | GTCAATGTTGCCCCCAGAGA | TTTTCGGAGCAGGAAAGCCC |
| GYS1 | CCGCTATGAGTTCTCCAACAAGG | AGAAGGCAACCACTGTCTGCTC |
| eNOS | TGATGGCGAAGCGAGTGAAG | ACTCATCCATACACAGGACCC |
| VE-cadherin | GTTCACCTTCTGCGAGGATATG | GATGGTGAGGATGCAGAGTAAG |
| 18-s | TGTGCCGCTAGAGGTGAAATT | TGGCAAATGCTTTCGCTTT |
